# Supplementary material for: Disseminated herpes simplex infection induced hepatitis during pregnancy mimicking HELLP syndrome, a diagnostic challenge
Source: Infect Med (Beijing). 2022 Jan 6;1(1):67–72. doi: 10.1016/j.imj.2021.12.001 (PMC10699672; doi:10.1016/j.imj.2021.12.001)
Supplement: Supplementary file 1 [file mmc1.docx]

**Article Title:** Disseminated herpes simplex infection induced hepatitis during pregnancy mimicking HELLP syndrome, a diagnostic challenge

**Abstract**:

The disseminated herpes simplex virus 2 (HSV-2) carries a high mortality rate in pregnant women if left unrecognized. It often presents as unrelieved fever and hepatitis. Diagnosis is challenging due to vague symptoms and potential overlap with other conditions. Pregnancy is a risk factor as it conforms to a partially immunocompromised state. Dissemination to the brain could be devastating, and the treatment requires intravenous antivirals like acyclovir. Fetal outcomes are variable based on previous case reports. We present a case of young female gravida 1 para 1 who presented with disseminated HSV infection mimicking HELLP (Hemolysis, Elevated Liver enzymes, and Low Platelets) syndrome. She responded well to intravenous acyclovir, and the fetus had a viable outcome at the 26th week of gestation. Early diagnosis can prevent progression to fulminant liver failure and the need for a liver transplant.

**Keywords**: Disseminated herpes simplex virus 2, Pregnancy, Infectious complications of pregnancy, HSV-2 infection, hepatitis in pregnancy

**Introduction**

In rare cases, the disseminated herpes simplex virus 2 (HSV-2) occurs in healthy patients, predominantly pregnant women. This could be secondary to the relative immunocompromised status induced by pregnancy. Six percent of reported cases occur in the third trimester, with a 50% risk of transplacental infection [1]. A high index of suspicion is required for pregnant women presenting with refractory pyrexia and hepatic dysfunction due to the mortality rates of disseminated HSV infection up to 50 % [2,3]. Presentation is varied, and treatment can result in up to 100% survival rates [2]. Disseminated HSV-2 hepatitis progresses to fulminant liver failure and mainly contributes to maternal mortality [3]. Acyclovir treatment significantly improves clinical outcomes for both fetus and mother if diagnosed early [3]. We report a case of the second trimester disseminated HSV-2 infection with hepatitis mimicking HELLP (Hemolysis, Elevated Liver enzymes, and Low Platelets) syndrome.

**Case presentation**

A 28-year-old female, at 26 weeks of gestation, was transferred to the medical intensive care unit with complaints of acute endometritis and intraabdominal bleeding. Her past medical history was significant for multiple miscarriages. She initially presented to OB/GYN service with high-grade fever, worsening flank pain, and concern for pyelonephritis. She was started on broad-spectrum antibiotics and supportive care but failed to improve. Her infectious workup did not show bacterial growth, and infectious disease suggested this was unlikely to be pyelonephritis. Her urinalysis was unequivocal for urinary tract infection and retroperitoneal ultrasonogram ruled out pyelonephritis.

Infectious disease (ID) further recommended checking Cytomegalovirus (CMV) IgM and IgG serology to rule out any other infectious etiology. As the patient had a kitten at home, they also recommended Toxoplasmosis IgM and IgG. Additional investigations included Monospot and viral respiratory panel.

Further history revealed that she was bitten by an insect over a week ago when she traveled to Washington DC. A physical exam revealed an erythematous circular pruritic lesion of 4 cm in diameter on the left thigh. Given new insect bite concerns, the patient was assessed for any tick-borne infection and tested for Arbovirus infection with acute arbovirus antibody test, Ehrlichia/Anaplasma Polymerase chain reaction (PCR), Rocky Mountain spotted fever, and Lyme titers.

The patient developed hypertension, elevated transaminases, low platelets, and down trending hemoglobin with schistocytes on peripheral blood smear. The trends of Lactate dehydrogenase (LDH), Alkaline phosphatase (ALP), Alanine transaminase (ALT), Aspartate transaminase (AST), and platelets are shown in figure 1 and 2. The following day (five days from admission), the patient had fluid leakage with Nitrazine test positive. In the setting of continuous fever and this episode, there was a concern for Premature rupture of membranes (PROM) and chorioamnionitis; the patient was started on latency antibiotics (ampicillin, gentamicin, and clindamycin), magnesium, and given betamethasone for fetal lung maturity. It was decided to deliver the baby at the 26th week of gestation via c-section. The patient received a magnesium infusion for 48 hours postpartum, and she continued to be febrile up to 101f and complained of abdominal pain, Computed Tomography Angiography (CTA) abdomen revealed hepatosplenomegaly with innumerable low-density liver lesions predominantly ranging from 2 to 5 mm (Figure 3a and 3b). Blood work for hepatitis was negative. The patient underwent a liver biopsy by interventional radiology that demonstrated microvesicular fatty infiltrate and moderate to severe microvesicular steatosis. ID on further follow-up recommended serology for Bartonella henselae due to the history of contact with the household kitten, and the patient was started on doxycycline. Two days after the biopsy (eight days from admission), she started complaining of abdominal and had a sharp drop of hemoglobin from 8g/dl to 5.5g/dl; urgent abdominal ultrasound demonstrating multiple liver lesions with mobile internal echoes suspicious for hemoperitoneum. Her repeat CTA of the abdomen was concerning for hemoperitoneum without active arterial extravasation, mottled appearance of the liver, and splenomegaly. The patient was transfused two units of packed red blood cells (PRBCs), one unit of fresh frozen plasma (FFP), and one unit of platelets. Acute care surgery and OB/GYN were consulted, and they deferred any surgery as no specific site of bleeding was identified on images. Her international normalized ratio (INR0 was 2.0, which would have only increased opportunities for additional bleeding. Her fibrinogen was normal (19mg/dl), D-dimers were elevated (96,781ng/ml), and the Prothrombin time was 22 seconds (INR 2.0).

Due to her elevated liver function tests and LDH, there was an additional concern for overlapping the acute fatty liver of pregnancy; gastroenterology recommended transfer to a center with transplant hepatology facilities 17 days after admission. A day later, the patient became acutely encephalopathic, tachycardic, and had new abdominal rebound tenderness concerning endometritis. A STAT CTA abdomen revealed intraperitoneal free fluid suspected to reflect hemoperitoneum increase in volume since the last exam. The patient was then transferred to the medical ICU on the 18th day of admission and started on broad-spectrum antibiotics (vancomycin and piperacillin-tazobactam). A magnetic resonance imaging (MRI) of the abdomen revealed a diffuse small nodular pattern of the liver parenchyma without hypervascular liver lesion and regenerating nodules of cirrhosis.

Given blood work and clinical symptomatology consistent with Disseminated intravascular coagulation (DIC) in the underlying HELLP syndrome and history of miscarriages, there was a concern for antiphospholipid syndrome (APS), catastrophic APS, or DIC. Antibodies were sent for anticardiolipin, anti-beta 2 glycoproteins, lupus anticoagulant on Hematology and/oncology consultation. They additionally recommended plasmapheresis given persistently worsening LDH, transaminases, and concern for catastrophic APS. Total bilirubin levels were normal (0.4 mg/dl). She received one session of plasma exchange. Her mental status continued to decline, and she was intubated for airway protection in the setting of encephalopathy. Her magnetic resonance imaging (MRI) showed symmetric restricted diffusion and increased T2/FLAIR signal within bilateral basal ganglia, which were nonspecific but suggested toxic, metabolic, or infectious encephalopathy.

The patient was also tested for Herpes simplex virus 1 and 2 as part of evaluation for a liver transplant for outside hospital transfer; On the 21st day of admission (fourth day in the ICU), Herpes simplex virus 2 (HSV-2) was detected in the patient's blood via real-time PCR targeting the glycoprotein B gene; And she was started on acyclovir 550mg every 8 hours and N-acetyl cysteine (NAC). She underwent a lumbar puncture, and Cerebrospinal fluid (CSF) was also positive for HSV-2, concerning HSV encephalitis. Her mental status later significantly improved with acyclovir, and she self-extubated a few days later. She dramatically improved on acyclovir and completed a 21-day high-dose course (IV 550mg every 8 hours) on the recommendation of Infectious Disease.

On subsequent follow-ups, she had normalization of her liver function (Figure 1 and Figure 2) and had no further complaints.

Of note, the newborn had an Apgar score of 1 min = 6 and 5 min = 8 with a birth weight of 880 grams. HSV 1 and 2 PCR were negative. Moreover, the hospital course of the newborn was complicated by respiratory distress syndrome, necrotizing enterocolitis, and Retinopathy of prematurity (zone II stage II). The newborn remained in the neonatal ICU for two months. After discharge, the newborn had appropriate growth/development with stable weight and good oral intake on a three-month follow-up.

**Discussion**:

Disseminated herpes simplex virus is a severe form of this viral illness that has been described in immunocompromised populations especially related to malignancy, medication-related immunosuppression, patients with bone marrow, and solid organ transplant recipients [5]. Herpes simplex virus has increased predominance in white females [4]. In pregnancy, possible immune modulation secondary to cell-mediated and humoral immunity alterations can increase the risk of disseminated herpes simplex virus infection [6]. Previously approximately 32 cases have been described of disseminated HSV infection in pregnancy, with the first case described in 1969 [7]. A literature search using PubMed database and keywords including “disseminated herpes simplex,” “HSV,” and pregnancy, combined with Boolean variables of ‘AND’ and ‘OR’ revealed seven case reports with adequate data regarding age of presentation, gestation, pertinent labs, management, and outcomes had been summarized in table 1.

Pregnant women with unexplained fever and suspicious history should be evaluated for disseminated HSV infection.

Females with primary HSV infection are more likely to develop the disseminated disease than secondary acquisition [7]. Due to the nature of recurrent illness and healing, lesions are detected in less than 50% of cases of HSV [8]. Previous reviews dictate that hepatitis is a common presentation in pregnant patients with disseminated HSV infection occurring up to 50% [4]. Hepatitis is the most common complication leading to coagulopathy and bleeding dyscrasias [4]. Common findings are that hepatosplenomegaly with jaundice, neurological symptoms, rash, liver dysfunction, and elevated inflammatory markers [3]. Reactive pyrexia and abnormal liver function tests should increase suspicion of possible disseminated HSV infection in pregnant females [2].

Diagnosis of disseminated herpes simplex virus infection in pregnancy is often challenging as history is often questionable, and lesions are missed on the clinical exam, if not widespread. Differential diagnosis includes fatty liver of pregnancy, disseminated intravascular coagulation, preeclampsia, or HELLP syndrome. Presentation is often in the third trimester, starting with anorexia malaise, fevers, myalgias; lesions are often missed as they occur less than 40% [2]. Other conditions associated with this disseminated infection include pericarditis, myocarditis, pancreatitis, and bone marrow suppression [4]. Treatment options include acyclovir, valacyclovir, and famciclovir [4]. Common findings seen in disseminated herpes simplex viral infection are included in table 2 [4]. Literature review reveals that the most common findings associated with disseminated HSV infection are constitutional symptoms and central nervous system changes. Abdominal pain, dysuria, urinary retention, and skin lesions occur less frequently [5]. Only 38% of immunocompromised patients had skin lesions, and abnormal liver function tests were found in 62% of patients [5]. Electroencephalogram (EEG) abnormalities may include abnormal patterns consistent with metabolic or toxic encephalopathy. In case of myocarditis, electrocardiogram (EKG) may show prolonged QRS, QT prolongation, diffuse T wave inversion, Ventricular arrhythmias, and atrioventricular conduction defects. High mortality and morbidity for mother and fetus remain a common prognosis if left untreated, with some studies describing mortality rate up to 50% [2,6]. Disseminated HSV infection has also been described previously with hemophagocytic syndrome during pregnancy resulting in inappropriately increased phagocytosis and negative outcome [2].

Our report demonstrates that treatment for disseminated HSV infections can improve maternal and fetal outcomes if diagnosed early. Differential diagnosis can be broad, and treatment often requires intravenous antivirals like acyclovir. Fetal outcomes are variable; however, further comparative data is needed to make definitive conclusions.

**Manuscript Word Count:** 1712 [excluding abstract and references]

**Table Count:** 2

**Figure Count:** 3

**Reference Count:** 11

**Data availability:** No data sets were used in this article.

**Author contributions:** **HA**: Conceptualization, Writing-Review & Editing, Supervision, Project Administration **SK**: Investigation, Resources, Writing-Original Draft, Visualization **MK**: Investigation, Resources, Writing-Original Draft, Proof reading **JK**: Investigation, Resources, Writing-Original Draft, Proof reading

**Competing interests:** The authors declare that they have no conflicts of interest.

**Acknowledgments:** We thank the patient for providing authorization for medical record use.

**References**:

1. Sauerbrei A, Wutzler P. Herpes simplex and varicella-zoster virus infections during pregnancy: current concepts of prevention, diagnosis, and therapy. Part 1: herpes simplex virus infections. Med Microbiol Immunol. 2007;196(2):89-94. doi:10.1007/s00430-006-0031-0
2. Goulding EA, Barnden KR. Disseminated herpes simplex virus manifesting as pyrexia and cervicitis and leading to reactive hemophagocytic syndrome in pregnancy. Eur J Obstet Gynecol Reprod Biol. 2014;180:198-9. doi:10.1016/j.ejogrb.2014.05.005
3. Bougioukas L, Psoinos RBC, Jones DC, Morris EA, Hale AJ. Disseminated herpes simplex virus 2 as a complication of pregnancy. IDCases. 2021;24:e01107. doi:10.1016/j.idcr.2021.e01107
4. Linthavong OR, Franasiak J, Ivester T. Febrile illness in pregnancy: disseminated herpes simplex virus. Obstet Gynecol. 2013;121(3):675-81. doi:10.1097/AOG.0b013e3182841753
5. Berrington WR, Jerome KR, Cook L, Wald A, Corey L, Casper C. Clinical correlates of herpes simplex virus viremia among hospitalized adults. Clin Infect Dis. 2009;49(9):1295-301. doi:10.1086/606053
6. Young EJ, Chafizadeh E, Oliveira VL, Genta RM. Disseminated herpesvirus infection during pregnancy. Clin Infect Dis. 1996;22(1):51-8. doi:10.1093/clinids/22.1.51
7. Hammad WAB, Konje JC. Herpes simplex virus infection in pregnancy - An update. Eur J Obstet Gynecol Reprod Biol. 2021;259:38-45. doi:10.1016/j.ejogrb.2021.01.055
8. Gupta R, Warren T, Wald A. Genital herpes. Lancet. 2007;370(9605):2127-37. doi:10.1016/S0140-6736(07)61908-4
9. Greenspoon JS, Wilcox JG, McHutchison LB, Rosen DJ. Acyclovir for disseminated herpes simplex virus in pregnancy. A case report. J Reprod Med. 1994;39(4):311-7.
10. Chazotte C, Andersen HF, Cohen WR. Disseminated herpes simplex infection in an immunocompromised pregnancy: treatment with intravenous acyclovir. Am J Perinatol. 1987;4(4):363-4. doi:10.1055/s-2007-999809
11. Hussain NY, Uriel A, Mammen C, Bonington A. Disseminated herpes simplex infection during pregnancy, rare but important to recognise. Qatar Med J. 2014;2014(1):61-4. doi:10.5339/qmj.2014.11

**Figure 1:** The trends of LDH, ALP, ALT, and AST throughout hospital course and on outpatient follow-ups, Red marker indicating ICU admission

LDH: Lactate dehydrogenase, ALP: Alkaline phosphatase, AST: Alanine transaminase, AST: Aspartate transaminase, ICU: Intensive care unit.

**Figure 2:** The trends of platelets throughout hospital course and on outpatient follow-ups, Red marker indicating ICU admission

ICU: Intensive care unit.

**Figure 3:** Computed Tomography Angiography (CTA) abdomen with low-density liver lesions, 3a (Coronal view) and 3b (Axial view)
